# Early ambulation protocol after diagnostic transfemoral cerebral angiography: an evidence-based practice project

**DOI:** 10.1186/s12883-024-03595-2

**Published:** 2024-03-25

**Authors:** Hao Liang, Richun Ye, Nana Song, Canhui Zhu, Miaolong Xu, Qiaoyu Ye, Lin Wei, Jiehan Chen

**Affiliations:** 1 Department of Neurology, the Second Affiliated Hospital of Guangzhou University of Chinese Medicine/Guangdong Provincial Hospital of Chinese medicine, Dade Road 111, Yuexiu District, Guangzhou510120, Guangdong China; 2 State Key Laboratory of Traditional Chinese Medicine Syndrome/Department of Nursing, the Second Affiliated Hospital of Guangzhou University of Chinese Medicine, 55 N, Neihuanxi Road, Guangzhou, 510006 Guangdong China

**Keywords:** Evidence-based practice, Cerebral angiography, Early ambulation

## Abstract

**Background:**

No uniform consensus has been achieved regarding the ambulation protocol after transfemoral cerebral angiography (TFA). Until now, in most hospitals patients are prescribed 8-12 h strict immobilization along with bed rest in the supine position after TFA in China, which causes great discomfort to patients.

**Objective:**

To evaluate the effect of an evidence-based early ambulation protocol on the prevention of vascular complications and general discomfort in patients following transfemoral cerebral angiography (TFA).

**Methods:**

A prospective quasi-experimental study was conducted on 214 patients undergoing TFA with manual compression. Patients in the experimental group were placed supine position for 2 h with a sandbag placed on the wound dressing, followed by a semi-seated position for another 2 h. After this period, patients took 2 h bed rest (move freely) with the sandbag removed, and were allowed to get out of bed 6 h after TFA. Patients in the control group were restricted to an 8 h bed rest in a supine position with the affected leg straight and immobilized. The vascular complications (bleeding, hematoma, ecchymosis) and levels of comfort (low back pain, leg pain, and blood pressure) were evaluated after the procedure. Numeric Rating Scale (NRS) pain scores, systolic blood pressure (SBP); diastolic blood pressure (DBP) were measured hourly for 8 h after TFA.

**Results:**

There was no significant difference in the two groups with regard to vascular complications including bleeding events (*P* = 0.621), bleeding volume (*P* = 0.321), and area of hematoma (*P* = 0.156).

The area of ecchymosis in the experimental group was significantly smaller than the control group (*P* = 0.031). Compared with the control group, the NRS score for low back pain in the 4th, 5th, 6th, 7th, and 8th hour after TFA were significantly lower (*P* < 0.05), and the NRS score for leg pain in the 5th, 6th, 7th, 8th hour after TFA were significantly lower (*P* < 0.05). The SBP and DBP in the 6th, 7th, and 8th hour after TFA were significantly lower than the control group (all *P* < 0.05).

**Conclusions:**

The evidence-based early ambulation protocol can effectively and safely increase comfort and decrease the pain level for patients undergoing TFA, without change in the incidence of vascular complications.

**Supplementary Information:**

The online version contains supplementary material available at 10.1186/s12883-024-03595-2.

## Introduction

Digital subtraction angiography (DSA) remains the gold standard for the diagnosis of cerebral vascular diseases, including the transfemoral or transradial artery approach [1]. Transfemoral cerebral angiography (TFA) is the predominant method in this test because of easy access, less contrast, and less radiation compared with the transradial approach [2, 3]. Reportedly, the most common complications associated with TFA are access site complications (e.g. arteriovenous fistula, pseudoaneurysm, retroperitoneal hematoma and thrombus, etc.), which have been reported in 1.96% (0.86–2.5%) of cases and usually occur within 12 h after TFA [4–6].

It is recommended that patients should receive 6–12 h of immobilization and bed rest in the supine position to prevent potential vascular complications following TFA [2, 7]. However, prolonged immobilization and bed rest inevitably lead to patient discomforts (low back pain, leg pain, etc.), difficulty defecation, and a longer hospital stay [4, 5]. In recent years, an increasing number of studies indicate that early ambulation accompanied by changing patients’ position after TFA could effectively reduce patient postoperative discomfort, without an increase in the vascular events [3, 8–10]. However, the early ambulation protocol varies among different studies, and no uniform consensus has been achieved regarding the duration of bed rest and optimal position after TFA. Until now, in most hospitals patients are prescribed 8-12 h strict immobilization along with bed rest in the supine position after TFA in China [2]. This controversial issue highlights the importance and the necessity of conducting an evidence-based practice project to summarize the best available evidence regarding the early ambulation protocol for TFA patients.

Therefore, this project aimed to institute an evidence-based early ambulation program in patients after TFA and evaluate its effectiveness and safety to best practice recommendations on nurse-led standardized postoperative management for patients undergoing TFA.

## Methods

### Design and context

The evidence-based team was built and responsible for the project implementation, monitoring, and evaluation. Team members included: two head nurses, five clinical nurses, and one neurologist. All members received systematically evidence-based training and have over three years of clinical experience. The Knowledge to Action (KTA) framework [11] provided overall guidance for this study. The KTA framework is one of the most frequently cited conceptual frameworks applied in the healthcare system to support knowledge creation and translation. This framework comprises two components: knowledge creation and an action cycle [12]. The methods for each of these two phases are described below.

### Project design: KTA knowledge creation

#### Knowledge Inquiry and Synthesist

A systematic literature search was conducted by a nurse specialist. The Population, Intervention, Comparison, Outcome (PICO) framework [13] was applied to organize the inclusion criteria: (1) participants undergoing angiography were included; (2) the intervention involves bed rest time or ambulation time; (3) the outcomes included vascular complications(bleeding, hematoma, or ecchymosis) or comfort measures (pain, vital signs). The search was carried out by the combination of medical subject headings (MeSH) and equivalent text word terms. The detailed research strategy in PubMed is presented in Table S1. Databases retrieved included PubMed, Science Direct, Web of Science, Cochrane Library, Up to Date, Chinese National Knowledge Infrastructure (CNKI), Wan Fang Data, Weipu (VIP) databases, National Institute for Health and Clinical Excellence(NICE), Scottish Intercollegiate Guidelines Network (SIGN), National Guideline Clearinghouse (NGC), Sigmarepository, and Nursing center.

After rigorous quality evaluation using the Appraisal of Guidelines, Research and Evaluation Instrument II (AGREE II) [14] and Joanna Briggs Institute Qualitative Assessment and Review Instrument (JBI-QARI), 12 articles were included (2 expert consensus, 6 random controlled trials. 2 evidence synthesis, 1 cohort trail, and 1 observational study) (see Table S2). The evidence-based team extracted evidence from included articles and formulated evidence-based knowledge for patients undergoing TFA, including 10 items covering four domains: closure approach, postoperative position management, immobilization and time in bed, and condition observation. The final evidence-based knowledge was evaluated and proven by consulting medical specialists and nurse specialists based on FAME (feasibility, appropriateness, meaningfulness, effectiveness) (Table S3).

### Project practice: KTA action cycle

#### Assessing barriers to knowledge use

In this research, root-cause analysis[15] was used to assess perceived barriers to the implementation evidence by the evidence-based team. Based on the root-cause analysis, the team members carried out the brainstorming to identify potential barriers in February 2022. The key aspects are summarized in three categories: innovations, potential adopters, and practice Environment. Based on the importance of research and feasibility to address, each barrier was rated using a 5-grade Likert scale by team members, ranging from one (not at all important/feasible) to five (extremely important/feasible). The identified barriers included:1) unfamiliar with the experimental protocol (importance, feasibility), 2) inertial thinking to previous protocol (importance, feasibility), and 3) Insufficient monitoring to implement change (importance, feasibility).

#### Selecting, tailoring, and monitoring

**Fig. 1:**
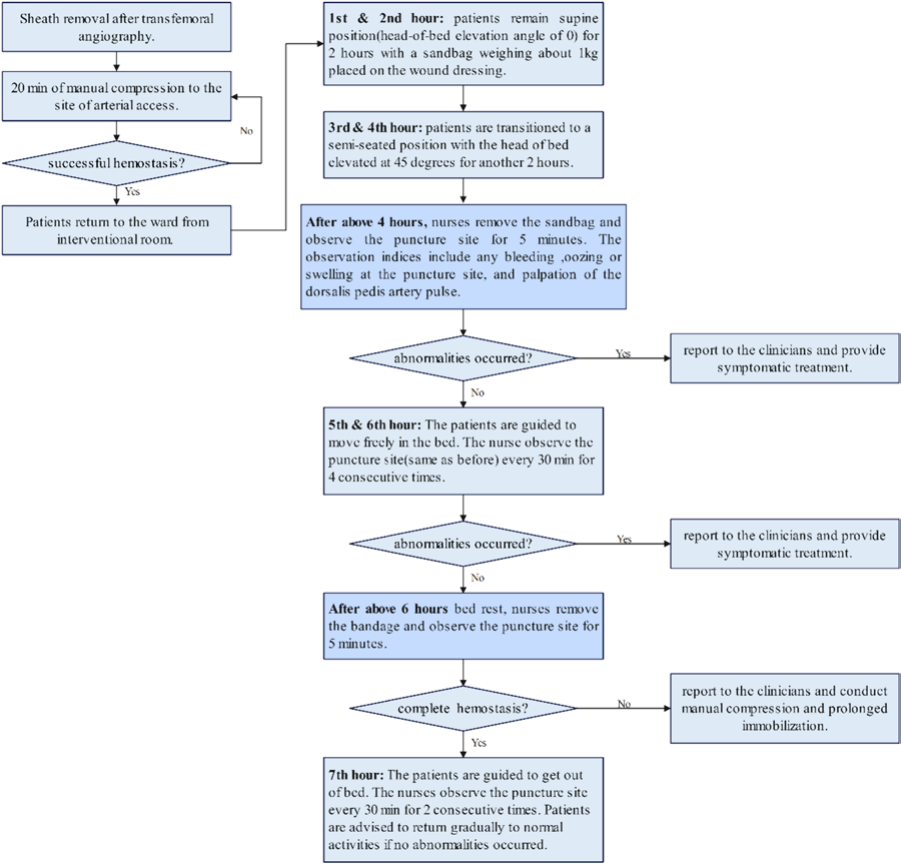
The workflow of Early ambulation protocol for patients after transfemoral angiography

Based on the top two barriers of “unfamiliar with the experimental protocol” and “inertial thinking to previous protocol”, the evidence-based team conducted two-week evidence-based program training for 23 clinical neurology nurses in February 2022. The training was conducted via three face-to-face discussions and online meetings, covering three modules: 1) introduction of the evidence-based program, 2) demonstration of the procedure, and 3) clinical examination of the procedure. A question and answer session followed the presentation in each module. Training materials including booklets and audio-visual materials were provided through the WeChat app. The workflow of Early ambulation protocol for patients after TFA was formulated based on evidence-based knowledge (see Fig. 1). As for another barrier “Insufficient monitoring to implementing change”, Audit-and-feedback [16] was conducted by the head nurse of the evidence-based team on surgery day (Tuesday and Friday). The Audit contents and methods were presented in Table S4.

#### Implementing intervention and evaluating outcomes

**Fig. 2:**
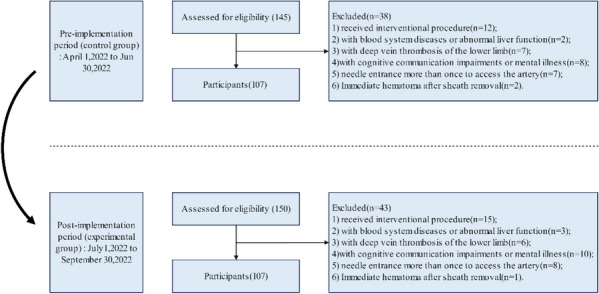
The workflow of the study

A prospective quasi-experimental study was conducted to evaluate the effectiveness and safety of an evidence-based early ambulation program, by comparing data from a historical control group (conventional protocol, from April 1, 2022, to Jun 30, 2022) with an experimental group (evidence-based protocol, from July 1, 2022, to September 30, 2022). The workflow of the study was presented in Fig. 2.

##### Participants

Participants involved in the project were recruited by convenience sampling. The inclusion criteria are outlined below: 1) candidate for diagnostic TFA under local anesthesia; 2) a sheath size was 5 or 6 French; 3) normal prothrombin time (PT) and international normalized ratio (INR) tests; 4) without severe bleeding or hematoma; 5) not received thrombolytic therapy; 6) informed consent. Exclusion criteria: (1) received interventional procedure; (2) vascular closure devices were used; (3) with blood system diseases or abnormal liver function; (4) with deep vein thrombosis of the lower limb; 5) with cognitive communication impairments or mental illness; 6) needle entrance more than once to access the artery; 7) Immediate hematoma after sheath removal.

##### Intervention

After the X-ray guided TFA procedure, all patients received standard vascular closure protocol, which consisted of 20 min of manual compression to the site of femoral artery puncture after sheath removal by an interventional radiologist. After the hemostasis ensued, 4 folded 4 × 4 gauzes were put on the femoral artery puncture to monitor for hematoma and bleeding, with a bandage covering the dressing.

After completion of the above periods, patients in the control group received conventional protocol. Patients were placed in a supine position for 8 h with a head-of-bed elevation angle of 0° after the TFA and were instructed to keep the punctured leg extended and immobilized. After 8 h of immobilization, patients were allowed to get up and sit at the side of the bed. If no palpable hematoma or active bleeding occurred, the bandage would be removed and patients were allowed to start ambulation.

In the experimental group, patients received the evidence-based program. Patients were asked to remain supine position for 2 h after completion of the manual compression period and a sandbag weighing about 1 kg was placed on the wound dressing. After 2 h of supine immobility, the patient was transitioned to a semi-seated position with the head of the bed elevated at 45 degrees for another 2 h. After this period, the sandbag was removed and the patients completed another 2 h of bed rest where they could move freely in the bed. After these 6 h in bed, patients were allowed to get out of bed if no complications were observed, with the bandage removed. Both groups receive a standardized postoperative care protocol in accordance with the Chinese expert consensus on the operating specifications of DSA [2].

##### Outcome measures

Demographic and medical history data were collected. Demographic characteristics (sex, age, body mass index, smoking, and alcohol history), conditions of combined underlying disease, history of antiplatelet use, indicators of coagulation function, and the surgery-related data (duration of surgery and sheath size).

Primary outcome measures: (1) Bleeding events and volume of bleeding: bleeding events are defined as any bleeding that requires manual compressions or surgical intervention [17]. Volume of bleeding was assessed by weighing the gauze before and after soaking it with blood [18]. (2) Area of hematoma and bruise: After gauze removal, the hematoma shape and bruise at the arterial puncture site were measured on a transparent paper, and the area was calculated by multiplying the longest and widest diameters. 3) Vascular complications: arteriovenous fistula, pseudoaneurysm, retroperitoneal hematoma, and lower-extremity deep vein thrombosis (DVT). All complications were recorded each hour and maintained for 12 h after TFA.

Secondary outcome measures:1) Numeric Rating Scale (NRS) pain scores: patients were required to describe pain intensity and sites using NRS ranging from 0 (no pain) to 10 (worst pain imaginable). NRS was measured hourly for 8 h after TFA by clinical nurses. 2) Vital signs: systolic blood pressure (SBP), diastolic blood pressure (DBP), and pulse rate was measured and recorded hourly for 8 h after TFA. Vital signs were measured using an electronic sphygmomanometer (Patent No.CN200930059890.3, China) by nurses.

### Statistical analysis

SPSS version 22.0 (IBM-SPSS Inc., Chicago, IL, USA) was applied for data analysis. Continuous data were tested for normality and are presented as means ± standard deviations (SD) or median and interquartile range as appropriate (normality distribution was tested with the Kolmogorov–Smirnov test). A two-tailed t-test was performed for normally distributed data, otherwise, Mann–Whitney U-test test was used. For categorical variables, frequencies and percentages were applied for data presentation. Pearson χ 2 tests and Fisher’s exact test were performed for data comparison. For repeated measures, one-way repeated-measures ANOVA was conducted to test the longitudinal changes in blood pressure between groups. A generalized estimation equation (GEE) was used to test the between- and within-group differences in NRS. the statistical significance level was set at the *P* < 0.05.

## Results

### Characteristics of patients

Between April 1, 2022, and September 30, 2022, a total of 214 participants were enrolled, with 107 patients in each group.127 males (59.3%) and 87 females (40.7%). The median age was years 64 (range 56–71) and the median BMI was 23.44 kg/m^2^ (range 21.21–25.95). The majority of patients (78.5%) were diagnosed with ischemic cerebrovascular disease. Most of the patients (78.0%) were on aspirin, clopidogrel, or both. There was no significant difference in the baseline parameters between the two groups (Table 1).

**Table 1:** Demographic Characteristics of the patients in two groups

Variable	Subgroup	Groups		*P* value
		Experimental *N* = 107	Control *N* = 107	
Age, years		63 (54,69)	67 (57,73)	0.064^a^
Sex	Male	60 (56.07)	67 (62.62)	0.330^b^
	Female	47 (43.93)	40 (37.38)	
Height(m)		1.62 (1.58,1.70)	1.65 (1.56,1.70)	0.673^a^
Weight(kg)		63.56 ± 11.13	62.78 ± 10.79	0.599^c^
BMI, kg/m2		23.70 ± 3.78	23.53 ± 3.53	0.732^c^
Drinking history	Has	11 (10.28)	14 (13.08)	0.523^b^
	Has not	96 (89.72)	93 (86.92)	
Smoking history	Has	34 (31.78)	30 (28.04)	0.550^b^
	Has not	73 (68.22)	77 (71.96)	
Disease diagnosis	Ischemic cerebrovascular disease	87 (81.31)	81 (75.70)	0.394^b^
	Hemorrhagic cerebrovascular disease	12 (11.21)	19 (17.76)	
	Others	8 (7.48)	7 (6.54)	
Charlson comorbidity index		1 (1,2)	1 (1, 2)	0.711^a^
Anticoagulant	None	23 (21.50)	24 (22.43)	0.980^b^
	Aspirin	15 (14.02)	13 (12.15)	
	Clopidogrel	24 (22.43)	25 (23.36)	
	Both	45 (42.05)	45 (42.06)	
Coagulation	PT	12.89 ± 0.86	12.96 ± 0.79	0.574^c^
	APTT	36.09 ± 4.32	36.05 ± 3.69	0.942^c^
	INR	0.98 (0.95,1.01)	0.96 (0.93,1.02)	0.163^a^
	FIB	3.42 (2.96,3.90)	3.29 (2.93,3.86)	0.216^a^
Duration of surgery, min		60 (48,62)	56 (45,61)	0.379^a^
Catheter size	5 F	99 (92.52)	100 (93.46)	0.789^b^
	6 F	8 (7.48)	7 (6.54)	

*Abbreviations: SD* Standard Deviation, *n* Number, *BMI* Body mass index, *PT* Prothrombin time, *APTT* Activated partial thromboplastin time, *INR* International standardized ratio, *FIB* Fibrinogen

^a^Mann-Whitney U test

^b^Pearson’s chi-squared test

^c^Independent t test

### Primary outcomes

Among the study participants, one and three patients experienced the bleeding event in the experimental and control groups, respectively. There was no statistical difference between groups in terms of the bleeding event (*P* = 0.621) and volume of bleeding (*P* = 0.321). Two cases in the control group developed hematoma in the femoral artery puncture site. No statistically significant differences were observed between groups in terms of hematoma size (*P* = 0.156). Notably, the area of ecchymosis in the experimental group was significantly smaller than in the control group (*P* = 0.031). No patients developed severe vascular complications, such as arteriovenous fistula, pseudoaneurysm, retroperitoneal hematoma, or lower extremity DVT (Table 2).

**Table 2:** Comparison of Complications after transfemoral angiography in two groups

Variable	Subgroup	Groups		* P* value
		Experimental *N* = 107	Control *N* = 107	
Bleeding event, n (%)	no	106 (99.07)	104 (97.20)	0.621^a^
	yes	1 (0.93)	3 (2.80)	
Time of bleeding(after surgery), h		0.00 (0.00, 0.00)	0.00 (0.00, 0.00)	0.318^b^
Amount of bleeding, ml		0.00 (0.00, 0.00)	0.00 (0.00, 0.00)	0.321^b^
Hematoma event, n (%)	no	107	105	0.498 ^a^
	yes	0	2	
Hematoma size, cm^2^		0.00 (0.00, 0.00)	0.00 (0.00, 0.00)	0.156^b^
Ecchymosis size, cm^2^		0.00 (0.00, 0.00)	0.00 (0.00, 0.00)	0.031^b^
Urinary retention, n (%)	no	104 (97.20)	98 (91.59)	0.075^c^
	yes	3 (2.80)	9 (8.41)	
urinary catheter, n (%)	no	106 (99.07)	101 (94.39)	0.119^a^
	yes	1 (0.93)	6 (5.61)	
Time of urinary catheter(after surgery), h		0.00 (0.00, 0.00)	0.00 (0.00, 0.00)	0.054^b^
Other vascular complications, n (%)	no	107 (100)	107 (100)	NA
	yes	0 (0)	0 (0)	

Other vascular complications include: arteriovenous fistula, pseudoaneurysm, retroperitoneal hematoma, and lower-extremity deep vein thrombosis (DVT)

*Abbreviation: NA* Not applicable

^a^Fisher’s exact test

^b^Mann-Whitney test

^c^Pearson’s chi-squared test

### Secondary outcomes

**Fig. 3:**
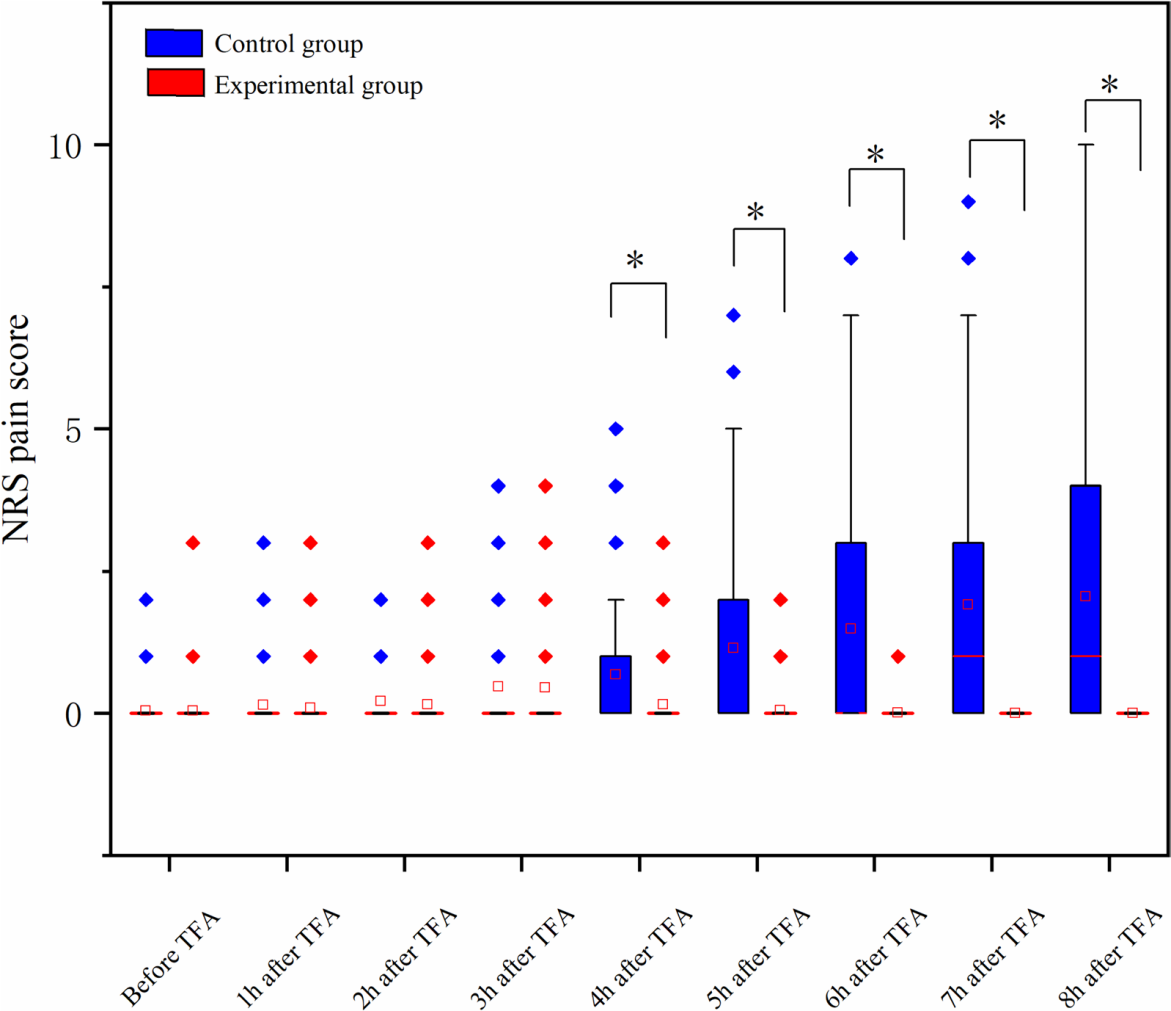
Comparison of low back pain scores between the two groups. NRS: Numeric Rating Scale. *P < 0.01

**Fig. 4:**
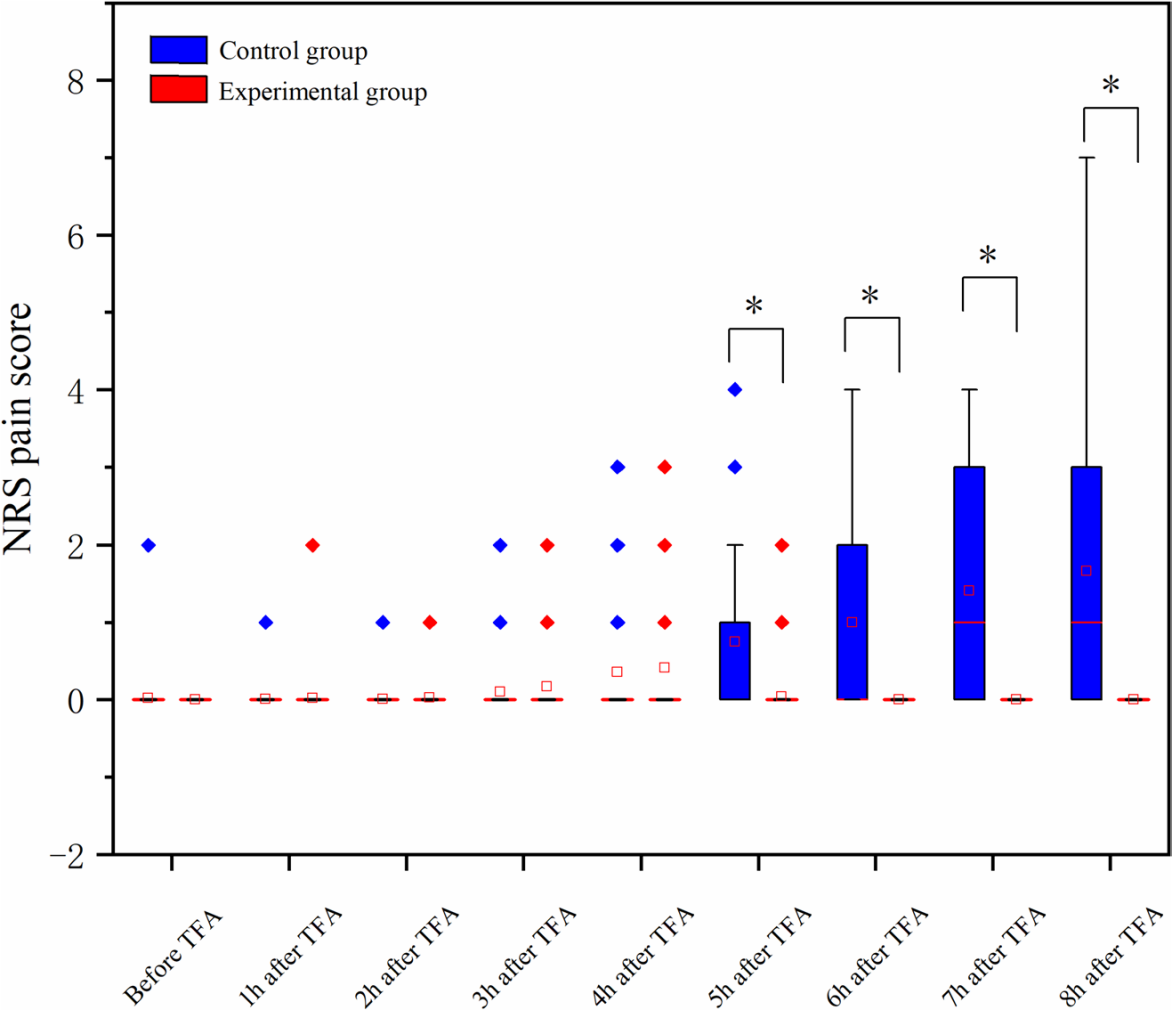
Comparison of leg pain scores between the two groups. NRS: Numeric Rating Scale. *P < 0.01

No significant difference was observed between the two groups in the NRS score of low back pain and leg pain before the TFA. There was a significant intergroup difference in NRS score for low back pain in the 4th, 5th, 6th, 7th, and 8th hour after TFA (all *P* < 0.05), with a lower score in the experimental group. With regard to the NRS score for leg pain, significant intergroup differences were not detected until the final 4 h (5th, 6th, 7th, and 8th hour after TFA) (all *P* < 0.05). The Generalized estimation equation (GEE) analyses showed statistically significant trends within the group in both NRS scores of low back pain (EG: Wald χ^2^ = 25.417, *P* < 0.0; CG: Wald χ^2^ = 91.022, *P* < 0.01) and leg pain(EG: Wald χ^2^ = 24.591, *P* < 0.01; CG: Wald χ^2^ = 101.073, *P* < 0.01) in two groups (Figs. 3 and 4; Table 3).

**Table 3:** Comparison of changes in patients’ pain scores in two groups

Variable	Time	Groups		* P* value
		Experimental *N* = 107	Control *N* = 107	
Low back pain	Before TFA	0.00 (0.00, 0.00)	0.00 (0.00, 0.00)	0.997^a^
	1st hour after TFA	0.00 (0.00, 0.00)	0.00 (0.00, 0.00)	0.415^a^
	2nd hour after TFA	0.00 (0.00, 0.00)	0.00 (0.00, 0.00)	0.617^a^
	3rd hour after TFA	0.00 (0.00, 0.00)	0.00 (0.00, 0.50)	0.612^a^
	4th hour after TFA	0.00 (0.00, 0.00)	0.00 (0.00, 1.00)	< 0.01^a^
	5th hour after TFA	0.00 (0.00, 0.00)	0.00 (0.00, 2.00)	< 0.01^a^
	6th hour after TFA	0.00 (0.00, 0.00)	0.00 (0.00, 3.00)	< 0.01^a^
	7th hour after TFA	0.00 (0.00, 0.00)	1.00 (0.00, 3.00)	< 0.01^a^
	8th hour after TFA	0.00 (0.00, 0.00)	1.00(0.00, 4.00)	< 0.01^a^
Between group (Wald χ^2^ = 53.109, *P* < 0.01)^b^		Wald χ^2^ = 25.417, *P* < 0.01^c^	Waldχ^2^ = 91.022, *P* < 0.01^d^	
Leg pain	Before TFA	0.00 (0.00, 0.00)	0.00 (0.00, 0.00)	0.317^a^
	1st hour after TFA	0.00 (0.00, 0.00)	0.00 (0.00, 0.00)	0.995^a^
	2nd hour after TFA	0.00 (0.00, 0.00)	0.00 (0.00, 0.00)	0.314^a^
	3rd hour after TFA	0.00 (0.00, 0.00)	0.00 (0.00, 0.00)	0.135^a^
	4th hour after TFA	0.00 (0.00, 0.00)	0.00 (0.00, 0.00)	0.758^a^
	5th hour after TFA	0.00 (0.00, 0.00)	0.00 (0.00, 1.00)	< 0.01^a^
	6th hour after TFA	0.00 (0.00, 0.00)	0.00 (0.00, 2.00)	< 0.01^a^
	7th hour after TFA	0.00 (0.00, 0.00)	1.00 (0.00, 3.00)	< 0.01^a^
	8th hour after TFA	0.00 (0.00, 0.00)	1.00 (0.00, 3.00)	< 0.01^a^
Between group (Wald χ^2^ = 61.830, *P* < 0.01)^b^		Wald χ^2^ = 24.591, *P* < 0.01^c^	Waldχ^2^ = 101.073, *P* < 0.01^d^	

*Abbreviations: NRS* Numerical Rating Scale, *TFA *Transfemoral coronary angiography

^a^Mann-Whitney U test was applied

^b^*P*-value of the comparison between experimental and control group in the analysis using generalized estimation equation

^c^*P*-value of the within experimental group in the analysis using generalized estimation equation

^d^*P*-value of the within control group in the analysis using generalized estimation equation

There was no significant difference in SBP, DBP, and pulse rate between the two groups before the TFA (all *P* > 0.05). The mean difference in SBP between groups was not statistically significant until the final 3 h (6th, 7th, and 8th hour after TFA) (all *P* < 0.05), with lower SBP in the experimental group. Similarly, there was a significant difference in DBP in the 4th and final three after the TFA between the two groups (all *P* < 0.05), with lower DBP in the experimental group. However, the pulse rate showed no significant difference between the two groups at each time point (all *P* > 0.05).

The change in SBP, DBP, and Pulse rate over time was analyzed using a One-way ANOVA.

A repeated-measures ANOVA of SBP revealed a significant decreasing trend in the experimental group (F = 2.132, *P* = 0.040) while a significant increasing trend was detected in the control group (F = 10.818, *P* < 0.001). As for DBP, a significant increasing trend in the control group (F = 4.323, *P* < 0.001) was observed while no significant trend of change was detected in the experimental group (F = 1.458, *P* = 0.182) (Table 4).

**Table 4:** Comparison of changes in patients’ blood pressure in two groups

Variable	Time	Groups		* P* value
		Experimental *N* = 107	Control *N* = 107	
Systolic blood pressure, mmHg	Before TFA	135.47 ± 19.75	133.03 ± 18.34	0.350^a^
	1st hour after TFA	131.21 ± 18.94	130.44 ± 19.02	0.765^a^
	2nd hour after TFA	131.59 ± 21.46	131.24 ± 18.58	0.900^a^
	3rd hour after TFA	133.70 ± 18.15	131.48 ± 22.64	0.429^a^
	4th hour after TFA	133.43 ± 18.04	134.57 ± 19.79	0.660^a^
	5th hour after TFA	133.35 ± 17.72	137.37 ± 20.94	0.130^a^
	6th hour after TFA	131.37 ± 17.44	140.26 ± 21.35	0.001^a^
	7th hour after TFA	131.40 ± 17.75	141.24 ± 21.00	0.000^a^
	8th hour after TFA	131.29 ± 18.11	143.87 ± 21.80	0.000^a^
Between group (F = 13952.528, *P* < 0.001)^b^		F = 2.132, *P* = 0.040^b^	F = 10.818, *P* < 0.001^b^	
Diastolic blood pressure, mmHg	Before TFA	77.21 ± 9.23	77.22 ± 10.89	0.989^a^
	1st hour after TFA	75.85 ± 9.39	76.70 ± 10.63	0.536^a^
	2nd hour after TFA	77.15 ± 8.91	77.07 ± 10.58	0.950^a^
	3rd hour after TFA	76.67 ± 8.49	78.21 ± 10.09	0.231^a^
	4th hour after TFA	75.63 ± 8.23	79.39 ± 10.01	0.003^a^
	5th hour after TFA	77.02 ± 9.17	79.58 ± 13.56	0.107^a^
	6th hour after TFA	75.79 ± 8.22	80.62 ± 11.58	0.001^a^
	7th hour after TFA	76 (70,80)	81 (76,87)	0.000^c^
	8th hour after TFA	74.88 ± 8.74	83.56 ± 12.14	0.000^a^
Between group (F = 22432.496, *P* < 0.001) ^b^		F = 1.458, *P* = 0.182^b^	F = 4.323, *P* < 0.001^b^	
Pulse rate	Before TFA	78 (71,82)	75 (68,82)	0.090^c^
	1st hour after TFA	76.51 ± 10.20	74.26 ± 10.12	0.106^a^
	2nd hour after TFA	75.21 ± 9.17	73.37 ± 10.07	0.166^a^
	3rd hour after TFA	72.80 ± 9.61	73.91 ± 9.45	0.398 ^a^
	4th hour after TFA	73.26 ± 8.70	73.46 ± 9.76	0.877^a^
	5th hour after TFA	73.67 ± 8.57	73.50 ± 10.75	0.899^a^
	6th hour after TFA	73.37 ± 8.48	73.41 ± 11.05	0.978^a^
	7th hour after TFA	72 (66,78)	73 (68,80)	0.412^c^
	8th hour after TFA	73.79 ± 9.18	74.67 ± 11.71	0.538^a^
Between group (F = 9188.417, *P* < 0.001)^b^		F = 3.523, *P* = 0.001 ^b^	F = 1.064, *P* = 0.395 ^b^	

*Abbreviations: SD*Standard deviation, *BPM* Beats per minute, *TFA* Transfemoral coronary angiography

^a^Independent t test

^b^Repeated measures analysis of variance

^c^Mann-Whitney U test

## Discussion

Based on the KTA framework, the current project institute an evidence-based early ambulation program in patients after TFA, including four dimensions: closure approach, postoperative position management, immobilization and time in bed, and condition observation. We further conduct a prospective quasi-experimental study to evaluate the effectiveness and safety of the early ambulation protocol, which find that compared to the conventional ambulation protocol, an evidence-based early ambulation program is effective and safe to reduce the severity of low back pain and leg pain without increasing the BP and the vascular complications.

With the rapid advancement in puncture technology, an increasing number of studies show that early ambulation and changing position in bed may increase patients’ comfort [4, 8]. A systematic review involving 4,019 patients found that a bed rest duration of 2–3 h after TFA can safely and effectively reduce back pain and discomfort with no change in the incidence of vascular complications [4]. However, the closure methods (such as arterial closure devices or manual compression) were not distinguished during data analysis in this review. A prospective observational study [10] demonstrated that for patients who received manual compression after TFA, the modified ambulation protocol including 4 h of immobilization and 6 h time in bed was safe and feasible. In addition to reducing ambulation time, changing patients’ position during immobilization was also proposed by previous research [3, 8, 19]. Two studies [3, 19] found that position change to a semi-seated position 2 h after TFA is effective and safe to reduce pain without increasing the vascular complications. Though many previous studies have demonstrated the benefits of early ambulation or position change, most protocols are single with the lack of systematization and wholeness (focused on early ambulation or position change only), and multiple interventions are generated based on experiences rather than scientific evidence. As in other countries, many hospitals in China are requiring patients to remain on prolonged bed rest from 8 to 12 h, with 8 h of immobilization after the procedure, which bring great discomfort to the patients.

The current project institute an evidence-based early ambulation program in patients undergoing TFA based on the Knowledge to Action model. The KTA model involves multiple stages, which may occur sequentially or simultaneously, or may overlap with different stages of the knowledge creation process [12]. In the current project, we successively conducted the four core stages of the KTA model: knowledge inquiry and synthesis, assessing barriers to knowledge use, selecting, tailoring, and monitoring, implementing the intervention and evaluating outcomes. Through these stages, we finally formulated and evaluated the evidence-based early ambulation protocol for patients undergoing TFA (See. Fig. 1). In the current early ambulation protocol, the supine position time, immobilization time, and bed rest time in the protocol were reduced from 8 h to 2 h, 4 h, and 6 h respectively. Similar to previous researches [3, 20], this study provides evidence that position change to a semi-seated position 2 h after TFA is effective and safe to reduce pain without increasing the vascular complications. Additionally, the current study provided new evidence that reduction of immobilization time and bed rest time to 4 and 6 h respectively were also feasible even if companies with the position change during immobilization, which is shorter than 6-8 h of immobilization in previous studies [3, 20].

The result showed that one (0.93%) and three patients (2.8%) experienced bleeding events in the experimental and control groups, respectively. A meta-analysis [4] of 20 studies found that 47 occurrences (2.2%) of bleeding after the transfemoral angiography was reported, which is comparable to our findings. Two patients in the control group developed hematoma in the femoral artery puncture site, while no case of hematoma was detected in the experimental group. More noteworthy is the significantly smaller area of ecchymosis in the experimental group than in the control group (*P* = 0.031). These may be associated with the application of a 1 kg sandbag which was placed on the wound dressing during the immobilization. There is controversy over the necessity, duration, and weight of keeping sandbags on catheter insertion. According to previous studies, a sandbag weighing 4kg placed on the wound dressing for 4-8 h was shown to be feasible and safe [3, 10, 19, 20]. The present study demonstrated the feasibility and necessity of using a sandbag weighing 1 kg on the catheter insertion for 4 h during immobilization. These results provide evidence to use sandbags reasonably in future ambulation protocols. There was no statistical difference between groups in terms of the bleeding event, the volume of bleeding, and hematoma or ecchymosis size, which well demonstrate the safety of the evidence-based early ambulation protocol.

The current study demonstrated that the NRS score for low back pain in the 4th, 5th, 6th, 7th, and 8th hour after TFA in the experimental group were significantly lower than the control group. The same difference was observed in the leg pain score in the final 4 h. These differences were mainly associated with the change of position (3rd h after TFA) and early bed mobility (5th after TFA) in the experimental group. Previous studies show that many complications may typically manifest following a prolonged period of bed rest, and the most common complications were low back pain and leg pain [8]. Hojjat et al. found that the low back pain and leg pain were reduced immediately with the position change to a semi-seated position from the 3rd hour after TFA [3]. These immediate effects were not observed in the current study, which may result from the fact that there was no pain or very mild pain during the first 3 h after TFA in both groups in this study (Figs. 3 and 4). Furthermore, the result presented that the early ambulation protocol could help to stabilize vital signs, evidenced by lower SBP and DBP in the final 3 h after TFA compared with the control group. However, no significant between-group difference was detected in blood pressure in another research [3]. The difference is mainly related to the 4 h of immobilization in the current study compared to 6 h of immobilization in previous research, which lead to lower pain scores in the experimental group in this study. Evidence suggests that an increase in blood pressure may indicate an increase in patients’ cortisol levels resulting from increased pain.

Overall, given the evidence in the current study that 4 h of immobilization and 6 h time in bed accompanied by changing position to a semi-recumbent position from the 3rd hour after TFA could reduce the severity of pain and stabilize blood pressure without an increase of vascular complication, it is highly recommended that patients following TFA receive an evidence-based early ambulation protocol to improve post-TFA comfort. There are several limitations in this study. Firstly, our study enrolled patients who planned to undergo diagnostic transfemoral angiography without intervention. Previous studies reported a higher complication rate in patients who undergo intervention, due to increased catheter and prolonged surgery time [21]. Therefore, our findings cannot be generalized to patients undergoing intervention. Secondly, patients with abnormal coagulation profiles were not included in this study considering the increased risk for hematoma or bleeding. It is recommended that this population is investigated in the future. Thirdly, though there were no increased cases of vascular complications due to early ambulation after TFA in both previous and present studies, we still need to be cautious in the use of the current evidence in clinical practice decisions, considering differences in operator experience, procedure performance, and other underlying conditions associated with the complications of the puncture site. Lastly, the randomized controlled trials (RCTs) design was not applied in this study due to the challenges of conducting different protocols in the same ward. We hope to conduct RCTs with follow-up in future studies to supplement our conclusions.

## Conclusions

Based on the results of this evidence-based practice project, we conclude that the early ambulation protocol including 4 h of immobilization and 6 h time in bed accompanied by changing position from the 3rd hour after TFA are related to increasing comfort and decreasing the pain level without increasing the vascular complications. This simple and relatively cost-free evidence-based early ambulation protocol can effectively improve the comfort of patients following TFA.

### Supplementary Information

**Supplementary Material 1.**

## Data Availability

Data are available from the corresponding author on reasonable request.

## Abbreviations

TFA: Transfemoral cerebral angiography
NRS: Numeric Rating Scale
SBP: Systolic blood pressure
DBP: Diastolic blood pressure
DSA: Digital subtraction angiography
KTA: Knowledge to Action
PICO: Population, Intervention, Comparison, Outcome
AGREE II: Appraisal of Guidelines, Research and Evaluation Instrument II
JBI-QARI: Joanna Briggs Institute Qualitative Assessment and Review Instrument
FAME: Feasibility, appropriateness, meaningfulness, effectiveness
PT: Prothrombin time
INR: International normalized ratio
DVT: Deep vein thrombosis
SD: Means ± standard deviations
GEE: Generalized estimation equation
BMI: Body mass index
RCTs: Randomized controlled trials
