# Supplementary material for: Early ambulation protocol after diagnostic transfemoral cerebral angiography: an evidence-based practice project
Source: BMC Neurol. 2024 Mar 25;24:104. doi: 10.1186/s12883-024-03595-2 (PMC10962163; doi:10.1186/s12883-024-03595-2)
Supplement: Supplementary file 1 — Supplementary Material 1. [file 12883_2024_3595_MOESM1_ESM.docx]

| **Table S1.**  **Search strategy for PubMed** | | |
| --- | --- | --- |
| Categories | Search terms | Results |
| Participant Condition | #1 "Angiography"[Mesh] | 261,804 |
|  | #2 ( "Angiography/methods"[Mesh] OR "Angiography/nursing"[Mesh] OR "Angiography/standards"[Mesh] ) | 51,387 |
|  | #3 Angiography[Title/Abstract] | 176,128 |
|  | #4  #1 or #2 or #3 | 340,844 |
|  | #5 "Femoral Artery"[Mesh] | 30,572 |
|  | #6 Femoral artery[Title/Abstract] | 20,237 |
|  | #7 Femoral arteries[Title/Abstract] | 4,458 |
|  | #8 Transfemoral[Title/Abstract] | 7,099 |
|  | #9  #5 or #6 or #7 or #8 | 47,994 |
| Intervention | #10 "Early Ambulation"[Mesh] | 3,217 |
|  | #11 ambulation[Title/Abstract] | 13,432 |
|  | #12 Mobilization[Title/Abstract] | 58,150 |
|  | #13 "Immobilization"[Mesh]  #14 Immobilization[Title/Abstract]  #15 "Bed Rest"[Mesh]  #16 Bed rest[Title/Abstract]  #17  #10 or #11 or #12 or #13 or #14 or #15 or #16  #18  #4 AND #9 AND #17 | 28,453  55,488  4,072  1,467  152,256  142 |

Execute Searches in PubMed:

https://pubmed.ncbi.nlm.nih.gov/?term=%28%28%28%28%22Angiography%22%5BMesh%5D%29+OR+%28%28+%22Angiography%2Fmethods%22%5BMesh%5D+OR+%22Angiography%2Fnursing%22%5BMesh%5D+OR+%22Angiography%2Fstandards%22%5BMesh%5D+%29%29%29+OR+%28Angiography%5BTitle%2FAbstract%5D%29%29+AND+%28%28%28%28transfemoral%5BTitle%2FAbstract%5D%29+OR+%28Femoral+arteries%5BTitle%2FAbstract%5D%29%29+OR+%28Femoral+artery%5BTitle%2FAbstract%5D%29%29+OR+%28%22Femoral+Artery%22%5BMesh%5D%29%29%29+AND+%28%28%28%28%28%28%28%22Early+Ambulation%22%5BMesh%5D%29+OR+%28ambulation%5BTitle%2FAbstract%5D%29%29+OR+%28Mobilization%5BTitle%2FAbstract%5D%29%29+OR+%28%22Immobilization%22%5BMesh%5D%29%29+OR+%28Immobilization%5BTitle%2FAbstract%5D%29%29+OR+%28%22Bed+Rest%22%5BMesh%5D%29%29+OR+%28Bedrest%5BTitle%2FAbstract%5D%29%29&sort=relevance&size=100

**Table S2 The characteristics of the included studies**

| Data source | Year | Country | The first author | Title | Study design |
| --- | --- | --- | --- | --- | --- |
| CNKI | 2018 | China | Neurological Society of Chinese Medical Association | Chinese expert consensus on operating specifications of digital subtraction  angiography. | Expert consensus |
| Up to Date | 2021 | America | Velagapudi | Preparing patients for cardiac catheterization and possible coronary artery intervention | Expert consensus |
| PUBMED | 2021 | Iran | Niknam | Comparison of the effectiveness of position  change for patients with pain and vascular  complications after transfemoral coronary  angiography: a randomized clinical trial | RCT |
| CNKI | 2021 | China | Guohua He | Study on the Methods of Compression Hemostasis and lower Limb  Immobilization after Cerebral Angiography | RCT |
| PUBMED | 2016 | Iran | Valiee | Evaluation of feasibility and safety of  changing body position after transfemoral  angiography: A randomized clinical trial | RCT |
| WEB OF SCIENCE | 2016 | Brazil | Matte | Reducing bed rest time from five to three hours does not increase complications after cardiac catheterization: the THREE CATH Trial1 | RCT |
| PUBMED | 2009 | Japan | Kato | Reduction of Bed Rest Time after Transfemoral  Noncardiac Angiography from 4 Hours to  2 Hours: A Randomized Trial and a One-arm Study | RCT |
| WEB OF SCIENCE | 2012 | America | Wilcoxson | Early Ambulation After Diagnostic Cardiac  Catheterization via Femoral Artery Access | RCT |
| PUBMED | 2014 | Iran | Mohammady | Early ambulation after diagnostic transfemoral catheterisation: a systematic review and meta-analysis | Systematic review |
| PUBMED | 2019 | Iran | Fereidouni | The efficacy of interventions for back pain  in patients after transfemoral coronary  angiography: A rapid systematic review | Systematic review |
| PUBMED | 2019 | America | Tonetti | Validation of an extrinsic compression and early  ambulation protocol after diagnostic transfemoral  cerebral angiography: a 5-year prospective series | Cohort study |
| CNKI | 2019 | China | Tianming Xu | Clinical observation of modified lower limb immobilized time after transfemoral cerebral angiography | Observational  Study |

**Table S3 Summary of evidence on early ambulation program in patients after transfemoral cerebral angiography**

| **Category** | **Evidence** |
| --- | --- |
| Closure approach | Manual compression is the standard for closing the femoral artery puncture site.^1,2^ |
|  | Manual compression for 10~20 min is recommended, until no active bleeding was observed at the wound site.^1,3-5^ |
|  | Vascular closure devices are recommended for patients who cannot cooperate with the immobilization protocol.^1^ |
| Position management | Changing patients’ position after transfemoral cerebral angiography are associated with decreasing the severity of back pain and improving patients’ comfort, without an increase in the vascular complications.^6-8^ |
|  | With a sandbag placed on the wound dressing, position change to a semi-seated position 2 hours after transfemoral angiography is effective and safe to reduce pain without increasing the vascular complications.^6,7^ |
|  | During the fifth and sixth hours after transfemoral angiography the patients are recommended to lie on the left or right side with the head angle of 15^0^.^7^ |
| Immobilization and time in bed | As for a sheath size of 4 or 5-French , standardized femoral closure strategy consisting of 20 minutes of manual compression followed by 2-4h ambulation with a sandbag placed on the wound dressing is shown to be feasible and safe.^6,8-12^ |
|  | Four hours of immobilization is recommended for patients undergoing transfemoral angiography with a 5F introducer sheath.^9^ |
|  | Patients who received manual compression can be ambulated and get out of bed after four to six hours.^2,9^ |
| Condition observation | Regularly observation for the puncture site (any bleeding ,oozing or swelling), and palpation of the dorsalis pedis artery pulse, and palpation of the dorsalis pedis artery pulse are recommended for early detection of vascular complications.^1,2^ |

**Table S4 The Audit contents and methods**

| Audit Items | Audit methods and subjects | Criteria for implementation |
| --- | --- | --- |
| **1st & 2nd hour:** After completion of the manual compression period, patients remain supine position(head-of-bed elevation angle of 0) for 2 hours and a sandbag weighing about 1kg was placed on the wound dressing. | Methods: field observation  Subjects: nurses and patients | 1) Patients are instructed to remain supine position for 2h±5min immediately after angiography with a sandbag placed on the dressing.  2) Patients are instructed to keep supine position with immobilization of the affected leg. |
| **3rd & 4th hour:** After 2 hours of supine immobility, patient was transitioned to a semi-seated position with the head of bed elevated at 45 degrees for another 2 hours. | Methods: field observation  Subjects: nurses | Patients are transitioned to a semi-seated position with the head of bed elevated at 45 degrees after 2h±5min of supine immobility. |
| After above 4 hours, nurses remove the sandbag and observe the puncture site for 5 minutes. The observation indices include any bleeding, oozing or swelling at the puncture site, and palpation of the dorsalis pedis artery pulse. The patients were guided to move freely in the bed if no abnormalities occurred. | Methods: field observation  Subjects: nurses and patients | 1) The sandbags are removed 4h±5min after angiography.  2) The patients are aware of the precautions of activities taken in bed. |
| **5th & 6th hour**: The nurse observed the puncture site(observation indices was the same as before) every 30 min for 4 consecutive times | Methods: field observation,  reviewing medical records(from PDA).  Subjects: nurses | The nurses observe the puncture site every 15min±5min for 4 consecutive times, and then again every 30min±5min for 2 consecutive times. |
| After above 6 hours bed rest, nurses remove the bandage and observe the puncture site for 5 minutes. The patients were guided to get out of bed if no abnormalities occurred. | Methods: field observation,  reviewing medical records(from PDA).  Subjects: nurses and patients | 1) Nurses remove the bandage 6h±10min after angiography.  2) The patients are aware of the precautions of activities out of bed. |
| **7th hour**: The nurses observed the puncture site every 30 min for 2 consecutive times. | Methods: field observation,  reviewing medical records(from PDA).  Subjects: nurses. | The nurses observe the puncture site every 30min±5min for 2 consecutive times. |

Abbreviation: PDA: personal digital assistant

**References**

1. Neurology branch of Chinese Medical Association. Chinese expert consensus on operating specifications of digital subtraction angiography. *Chin J Neurol*. 2018;51:7-13.

2. Velagapudi P. Preparing patients for cardiac catheterization and possible coronary artery intervention. In: UP to DATE; 2022.

3. Tonetti DA, Ferari C, Perez J, Ozpinar A, Jadhav AP, Jovin TG, Gross BA, Jankowitz BT. Validation of an extrinsic compression and early ambulation protocol after diagnostic transfemoral cerebral angiography: a 5-year prospective series. *J Neurointerv Surg*. 2019;11:837-840. doi: 10.1136/neurintsurg-2018-014572

4. HE Guohua, Zhen W. Study on the Methods of Compression Hemostasis and lower Limb Immobilization after Cerebral Angiography. *Clinical Research*. 2021;29:31-33.

5. Matte R, Hilario Tde S, Reich R, Aliti GB, Rabelo-Silva ER. Reducing bed rest time from five to three hours does not increase complications after cardiac catheterization: the THREE CATH Trial. *Rev Lat Am Enfermagem*. 2016;24:e2796. doi: 10.1590/1518-8345.0725.2796

6. Valiee S, Fathi M, Hadizade N, Roshani D, Mahmoodi P. Evaluation of feasibility and safety of changing body position after transfemoral angiography: A randomized clinical trial. *J Vasc Nurs*. 2016;34:106-115. doi: 10.1016/j.jvn.2016.05.001

7. Rezaei-Adaryani M, Ahmadi F, Asghari-Jafarabadi M. The effect of changing position and early ambulation after cardiac catheterization on patients' outcomes: a single-blind randomized controlled trial. *Int J Nurs Stud*. 2009;46:1047-1053. doi: 10.1016/j.ijnurstu.2009.02.004

8. Niknam Sarabi H, Farsi Z, Butler S, Pishgooie AH. Comparison of the effectiveness of position change for patients with pain and vascular complications after transfemoral coronary angiography: a randomized clinical trial. *BMC Cardiovasc Disord*. 2021;21:114. doi: 10.1186/s12872-021-01922-w

9. Xu Tianming, Cai Dongyang, Xue Jiangyu, Yang Bowen, Shi Weiyu, Zhao Tongyuan, Xu Gangqin, Kang Xiaoyu, He Yingkun, Xu Bin, et al. Clinical observation of modified lower limb immobilized time after transfemoral cerebral angiography. *Chin J Inter Rad（Electronic Edition）*. 2019;7. doi: 10.3877/cma.j.issn.2095-5782.2019.04.005

10. Kato F, Sato Y, Yuasa N, Abo D, Sakuhara Y, Oyama N, Onimaru R, Aoyama H, Shirato H, Terae S. Reduction of bed rest time after transfemoral noncardiac angiography from 4 hours to 2 hours: a randomized trial and a one-arm study. *J Vasc Interv Radiol*. 2009;20:587-592. doi: 10.1016/j.jvir.2009.02.003

11. Wilcoxson VL. Early Ambulation After Diagnostic Cardiac Catheterization via Femoral Artery Access. *The Journal for Nurse Practitioners*. 2012;8:810-815. doi: 10.1016/j.nurpra.2012.06.002

12. Mohammady M, Heidari K, Akbari Sari A, Zolfaghari M, Janani L. Early ambulation after diagnostic transfemoral catheterisation: a systematic review and meta-analysis. *Int J Nurs Stud*. 2014;51:39-50. doi: 10.1016/j.ijnurstu.2012.12.018
